# CISNE: An accurate description of dose-effect and synergism in combination therapies

**DOI:** 10.1038/s41598-018-23321-6

**Published:** 2018-03-21

**Authors:** Amador García-Fuente, Fernando Vázquez, José M. Viéitez, Francisco J. García Alonso, José I. Martín, Jaime Ferrer

**Affiliations:** 1 Departamento de Física, Universidad de Oviedo, Oviedo, E-33007 Spain; 2 Oncology University Institute of the Principality of Asturias (IUOPA), Oviedo, 33006 Spain; 3 Departamento de Oncología Médica, Hospital Universitario Central de Asturias, Oviedo, E-33011 Spain; 4 Departamento de Química Orgánica e Inorgánica, Universidad de Oviedo, Oviedo, E-33006 Spain; 5 Research Center for Nanomaterials and Nanotechnology (CINN, CSIC), El Entrego, E-33940 Asturias, Spain

## Abstract

The precise determination of dose-effect curves and the combination effect of drugs is of crucial importance in the development of new therapies for the most dreadful diseases. We have found that the current implementations of the theory of Chou *et al*. are not accurate enough in some circumstances and might lead to erroneous predictions of synergistic or antagonistic behaviour. We have identified the source of inaccuracies and fixed it thereby improving the accuracy of those methods. Here we explain the main features of our approach and demonstrate its higher accuracy as compared to the standard methods. Therefore, this new implementation might have a huge impact in the reliability of future research on new Combination Therapies.

## Introduction

Combination Therapies have been a valuable approach for the treatment of diseases since the origins of medicine. Traditional Chinese medicine has been using mixtures of natural herbs for more than 2000 years^1^. Nowadays, the use of adequate drug combinations allows to increase beneficial effects using lower doses of each constituent and therefore to reduce their adverse effects, as well as to minimize the induction of drug resistance^2^. It can also provide selective synergism against a particular target. For these reasons, Combination Therapies are widely used for the treatment of the most dreadful diseases, such as cancer^3,4^ or AIDS^5,6^.

Potential drug combinations are frequently tested first in cell cultures in preclinical environments, whereby the successful combinations are subsequently transferred to animal experimentation, clinical testing and clinical practice. However, the transition from a biologically controlled environment to the clinical practice often results in failures^7,8^. This lack of success is possibly caused by the difficulty to reproduce the overall physiological conditions in the laboratory. However, they would also occur if both drugs act on the same cellular sub-clone. We finally note that some of those failures might be originated by an insufficiently accurate statistical analysis of the cell cultures that may provide false positives or negatives in specific situations. The goal of the present article is to eliminate this last source of errors by implementing drastic improvements of the referred statistical analysis

**Figure 1:**
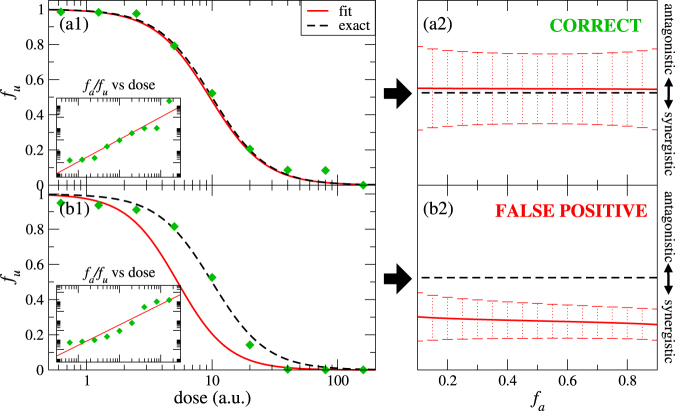
Example of a dose(in arbitrary units a.u.)-effect(unaffected fraction *f*_*u*_) data (green diamonds) for which currently available methods (red lines) reproduces accurately the shape of the dose effect curve (panel (a1)) or completely fails to reproduce it (panel (b1)). This can lead to a correct determination of the synergism (panel (a2)) or to a completely wrong result (panel (b2)). The exact result (black dashed lines), obtained from exact data previous to introduce a experimental deviation, is also shown. Insets of panels (a1) and (b1) show the linear regression used to fit the data.

Computational methods can be applied to assist on the determination of the effect of Combination Therapies. First, they can be used in an effort to predict, previous to experimental verification, potential drug combinations^9–11^. Also, they can be used after experiments to analyse statistically the measured data in order to quantify the presence or absence of synergism^12,13^, that is the subject matter of the present work. Currently available methods^12,13^ to quantify the synergistic effect of drug combinations from experimental data predict accurate dose-effect relationships in many cases (Fig. 1(a1)), which lead to a correct prediction of the synergism results (Fig. 1(a2)). However, we have found that they fail to reproduce the experimental data in quite a few others (Fig. 1(b1)). This lack of precision may result in an inaccurate or even erroneous description of the synergistic properties of drug combinations (Fig. 1(b2)). Therefore, there is an urgent need to develop a method that improves the accuracy of the dose-effect description, which then enables us to identify synergism effects in experiments with a minimal uncertainty.

Although drug synergism might seem an intuitive concept, its formal definition is not so simple and it has been a focus of controversy during the past century^14–16^. A combination of drugs is synergistic when the combined effect is larger than the additive effect of each individual drug. Similarly, the combination is antagonistic when its combined effect is smaller than the additive effect of each individual drug. Trouble arises however from the definition of the additive effect. Let us assume that two drugs A and B have an effect that we quantify with the numbers *E*_A_ and *E*_B_ (e.g.: the number of affected cells in a system). We define tentatively the additive effect of the two drugs as *E*_A_ + *E*_B_. However, this definition would be true only if the dose-effect relationships of each drugs were linear, which is usually not the case. The modern definition of drug synergism^16–21^ is based on the work by Chou *et al*., who define the median-effect equation (MEE)^22–24^ as the universal equation for the non-linear dose-effect relationship. This definition is based on physicochemical processes, and its validity does not depend upon factors that are specific of each system such as the mechanism of the enzymatic reaction or the kinetic nature of the inhibitor. From that, they define the Combination Index (*CI*)^25,26^ to quantify the synergistic effect of a drug combination. A given drug combination is said to be additive when its *CI* equals 1. The degree of synergism (or antagonism) is measured by the deviation of *CI* from 1, the smaller (the larger) the *CI*, the stronger the synergistic (antagonistic) effect of the combination is. Therefore, in order to analyse synergism in a drug combination, a full and accurate description of the dose-effect relationship of each drug separately, as well as of their combination at a fixed drug ratio, is needed^16^.

In this work, motivated by our need to obtain results as accurate as possible from synergism studies, we have analysed the numerical problems of the currently available methods such as calcusyn^12^ and compusyn^13^. We present our new implementation of the theory of Chou *et al*., which solves these problems, thus increasing the precision and reliability of previous methods. We have made the new implementation freely available through the use of our new program CISNE (Code for the Identification of Synergism Numerically Efficient)^27^. Therefore, our new method keeps the generality of a theory that has been widely validated and applied to a variety of biological systems^28^, but, at the same time, it represents an improvement on the implementation of that theory. This is because it allows to determine accurately possible synergistic effects in biological systems within a wide range of characteristic parameters where the previous methods failed.

## The theory of synergism

**Figure 2:**
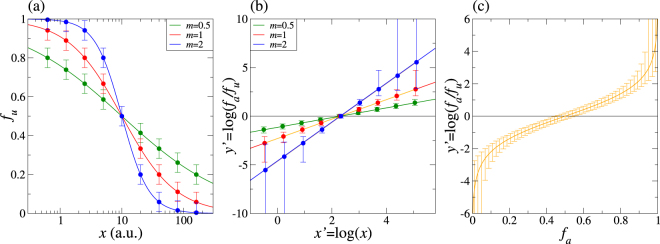
(**a**) Dose-effect relationships in terms of a median effect equation with *D* = 10 and different values of *m*. Dose values *x* and *D* are given in arbitrary units. Theoretical data points with a fixed standard deviation σ(*f*_*a*_) = 0.05 are also included. (**b**) Linear fitting variables *x*′ and *y*′ plotted using the same data of panel (a). (**c**) Dependence of *y*′ and of its standard deviation σ(*y*′) with the value of *f*_*a*_ for a fixed value of σ(*f*_*a*_) = 0.05.

In a dose-effect experiment several doses *x* of a drug are applied to a population (e.g.: a cell culture), and their effect is determined by measuring the fraction of the population affected by the drug after a certain period of time, *f*_*a*_. Current practice actually plots the fraction unaffected *f*_*u*_ = 1 − *f*_*a*_, as a function of *x*, as we show in Fig. 1. Based on physicochemical analyses, Chou *et al*. encapsulated the dependence of *f*_*a*_ on the dose in terms of the following non-linear universal equation (MEE)^22,23^:

1 $${f}_{a}/{f}_{u}={(x/D)}^{m}$$

where *D* and *m* are parameters that depend on each given drug. *D* is the dose at which *f*_*a*_ = 0.5, whereas *m* determines the shape of the dose-effect curve. Here *m* = 1, >1 or <1 indicate hyperbolic, sigmoidal or flat sigmoidal curves, respectively. Examples of these different curves are given in Fig. 2(a).

Synergism is characterized by the combination index *CI*, that is always given as a function of *f*_*a*_. We discuss in some detail here the combination index for two drugs, although the formalism can be extended for N drugs. The combination index for drugs A and B combined in a ratio R _*A*_:R _*B*_ is given by

2 $$CI({f}_{a})={x}_{A}^{C}/{x}_{A}^{0}+{x}_{B}^{C}/{x}_{B}^{0}$$

where ${x}_{A,B}^{0}$ and ${x}_{A,B}^{C}$ are the doses of drug *A* or *B* that produce an effect *f*_*a*_ alone and in the combination, respectively. Solving for *x* in Eq. (1) yields *x* = *D*(*f*_*a*_/*f*_*u*_)^1/*m*^. This expression for A, B and A + B can be inserted into Eq. (2) yielding

3 $$CI({f}_{a})=\frac{{D}_{A+B}}{{R}_{A}+{R}_{B}}\,{(\frac{{f}_{a}}{{f}_{u}})}^{\frac{1}{{m}_{A+B}}}(\frac{{R}_{A}}{{D}_{A}}{(\frac{{f}_{u}}{{f}_{a}})}^{\frac{1}{{m}_{A}}}+\frac{{R}_{B}}{{D}_{B}}{(\frac{{f}_{u}}{{f}_{a}})}^{\frac{1}{{m}_{B}}})$$

where *m*_*A*,*B*,*A*+*B*_, *D*_*A*,*B*,*A*+*B*_ are the MEE parameters obtained for drugs *A* or *B* alone or in the combination *A* + *B*. Notice that a not sufficiently accurate determination of any of these parameters may lead to erroneous estimates of *CI*.

We discuss now how popular implementations of Chou’s theory^12,13^ determine the important parameters *m* and *D* of a given drug and give some hints on the source of their inaccuracies. First, Eq. (1) is converted to the linear relationship.

4 $$y^{\prime} =mx^{\prime} +b$$

where *y*′ = log(*f*_*a*_/*f*_*u*_), *x*′ = log(*x*) and *b* = −*m*log(*D*). Then the experimental data points are fitted to Eq. (4) using a linear least squares fit. This procedure delivers estimates for *m* and *b*. The remaining parameter is obtained from *D* = *e*^−*b*/*m*^. This approach is accurate whenever the experimental data points obey closely the theoretical dose-effect curves. In practice, the experimental dose-effect data obtained from biological samples always contain some non negligible dispersion whose impact need not be negligible.

## Why current implementations fail

We illustrate the impact of actual experimental data dispersion via an example. Imagine an experiment in a culture where the measured fraction unaffected of the population *f*_*u*_ leads to the any of the curves plotted in Fig. 2(a). Here we give the same standard deviation σ to each experimental data point. We plot now the same data points in a logarithmic scale in Fig. 2(b), so that the axes represent *x*′ and *y*′. The figure demonstrates that a constant σ for *f*_*u*_ leads to a non constant standard deviation σ′ for *y*′. Importantly, *σ*′ increases hugely at the sides of the curve specially if *m* is sufficiently large. This pathology is made more evident in Fig. 2(c), where we show how *y*′ and σ′ depend on *f*_*a*_. The figure shows that σ′ increases and eventually diverges as we move away from *f*_*a*_ = 0.5. This means that the linear least squares fit procedure gives artificially more weight to those experimental points having *f*_*a*_ closer to 0 or 1. Therefore, the results are artificially biased. We will show below that this artifact can harm substantially the quality of the results even leading to false positives or false negatives verdicts of synergistic behaviour.

Our new implementation in CISNE avoids the above pathology from the outset. We take advantage of modern curve fitting algorithms, that obtain *m* and *D* directly from a nonlinear least squares fitting^29^ of equation (1). This approach presents two main advantages:

1. All the data points share the same weight, that is independent of the value of *f*_*a*_ thus avoiding any bias.
2. Experimental data points having *f*_*a*_ ≥ 1 or *f*_*a*_ ≤ 0 happen experimentally due to experimental fluctuations and carry information that should not be ignored. The linear least squares fitting does ignore them and misses relevant information while the nonlinear least squares fitting does not present this limitation, and therefore no data information is lost.

## A false positive and a false negative

**Figure 3:**
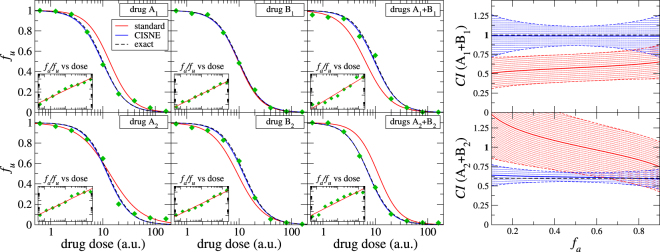
Dose-effect fitting and combination index *CI* obtained for a theoretical additive combination A_1_ + B_1_ (upper panels) and a theoretical synergistic combination A_2_ + B_2_, as described in the text, with the standard method (red lines) and with the new CISNE method (blue lines), compared to the exact result (black dashed lines). Each dose-effect panel includes an inset with the result of the linear regression used by the standard method. Blue and red shaded regions in the *CI* indicate the 95% confidence interval of the results obtained with the standard method and with CISNE, respectively.

We show now two examples where the current implementation fails while CISNE reproduces the correct behaviour. In the first example, two drugs A_1_ and B_1_ show purely additive behaviour (*CI* = 1), however the current implementation predicts strong synergism ($CI\ll 1$). We generate dose-effect data points numerically for the two drugs and for their combination A_1_ + B_1_ in a 1:1 ratio, that we show in the upper left panel in Fig. 3. Notice that we have introduced random deviations in the data points following a standard deviation σ = 0.05 to try and mimic experimental data. The generated dose-effect data is given in Table 1 of the Additional Information. The upper left panel of Fig. 3 shows the results of the dose-effect fitting curve with the standard (red lines) and the CISNE (blue lines) methods, as well as the exact curve before introducing the experimental deviations (black dashed lines). The panel shows that the current implementation reproduces well some of the dose-effect relationships but it fails for others, while CISNE fits accurately the experimental data points. More important however are the predictions for the combination index obtained from the fitted curves, that are shown in the upper right panel of the figure, together with their 95% confidence interval. Here CISNE reproduces quite correctly the additive behaviour of the combination while the standard method predicts a strong synergistic effect leading to a false positive.

**Table 1:** Generated dose *x* and fraction affected *f*_*a*_ data of a combination of drugs A_1_ + B_1_ with additive behavior.

drug A_1_		drug B_1_		drug combination A_1_ + B_1_	
*x*	*f*_*a*_	*x*	*f*_*a*_	*x*	*f*_*a*_
0.625	0.999	0.625	0.997	0.625	0.956
1.25	0.992	1.25	0.970	1.25	0.933
2.5	0.963	2.5	0.959	2.5	0.919
5	0.789	5	0.782	5	0.745
10	0.469	10	0.484	10	0.559
20	0.182	20	0.221	20	0.134
40	0.115	40	0.045	40	0.047
80	0.046	80	0.009	80	0.003
160	0.012	160	0.005	160	0.003

In the second example, two drugs A_2_ and B_2_ show a strongly synergistic behaviour (*CI* = 0.6). We generate dose-effect data points for the two drugs and for their combination A_2_ + B_2_ in a 1:1 ratio, where we include random deviations as before. The dose-effect curves are plotted in the lower left panel of Fig. 3 and the corresponding data are given in Table 2 of the Additional Information. The panel also shows the curves fitted with the standard method as well as with CISNE. As in the former example, we find that CISNE fits the experimental data points much more accurately. We use these curves to determine the combination index. We plot our results in the lower right panel of Fig. 3 together with their 95% confidence interval. We find that CISNE reproduces accurately the synergistic behaviour while the standard method predicts additivity or even antagonism leading to a false negative.

**Table 2:** Generated dose *x* and fraction affected *f*_*a*_ data of a combination of drugs A_2_ + B_2_ with synergistic behavior.

drug A_2_		drug B_2_		drug combination A_2_ + B_2_	
*x*	*f*_*a*_	*x*	*f*_*a*_	*x*	*f*_*a*_
0.625	0.992	0.625	0.988	0.625	0.999
1.25	0.964	1.25	0.951	1.25	0.998
2.5	0.923	2.5	0.924	2.5	0.911
5	0.838	5	0.828	5	0.673
10	0.648	10	0.624	10	0.365
20	0.202	20	0.263	20	0.104
40	0.107	40	0.054	40	0.037
80	0.067	80	0.020	80	0.020
160	0.058	160	0.005	160	0.018

Notice that the wrong results with the standard method are a consequence of the inaccurate description of some of the dose-effect data by the fitted curves, especially in the central range of *f*_*u*_, which is not visible in the linear fittings shown in the insets of Fig. 3 (where r > 0.96 in all cases indicating an accurate linear fitting). In the first example the fitted curve underestimates the effect of drug A_1_ (the fitted curve is shifted to the right respect to the dose-effect data points), but it overestimates the effect of the drug combination A_1_ + B_1_ (the fitted curve is shifted to the left respect to the dose-effect data points). This combined effect leads to a large overestimation of the effect of the combination of drugs A_1_ + B_1_ respect to their individual effect, and in this case to a false positive. Similarly, in the second example the effect of drug B_2_ is overestimated and the effect of the drug combination A_2_ + B_2_ is underestimated, leading to a large underestimation of the effect of the combination of drugs A_2_ + B_2_ respect to their individual effect, and, therefore, to a false negative in this case.

## More computational experiments

In order to evaluate the precision of the new methodology implemented in CISNE compared to the standard procedure more rigorously, we have analysed the MEE parameters and *CI* obtained from experimental data generated computationally by means of Monte Carlo simulations^30,31^. In these simulations we consider the data points shown in Fig. 2(a), which for the parameters *D* and *m* lead to expected values of the parameters E[*D*] = 10 (in arbitrary units) and E[*m*] = 0.5, 1, 2. The generated data includes 9 different drug concentrations, including *x* = *D* and 4 concentrations above and below *D*, with 2 adjacent concentrations differing in a factor of 2. For each data point, a randomly generated error is included in *f*_*a*_ following a normal distribution with a standard deviation σ(*f*_*a*_) = 0.05. From these generated data for each curve the values of *D* and *m* are recalculated with both the standard and the new CISNE methods, and compared to their expected values E[*D*] and E[*m*]. Standard deviations σ(*D*) and σ(*m*) are also calculated from the fitting procedures. Each computational experiment is repeated 100000 times to obtain statistical significant results.

**Figure 4:**
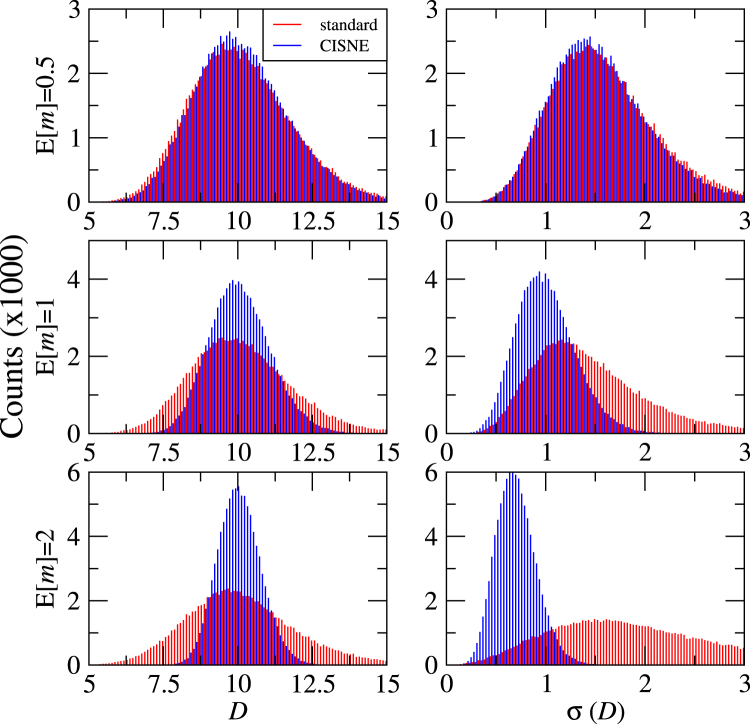
Results of 100000 Monte Carlo simulations for the parameter *D* and its standard deviation σ(*D*), obtained with the standard procedure and with the new method implemented in CISNE, for curves of different expected sigmoidality E[*m*].

**Figure 5:**
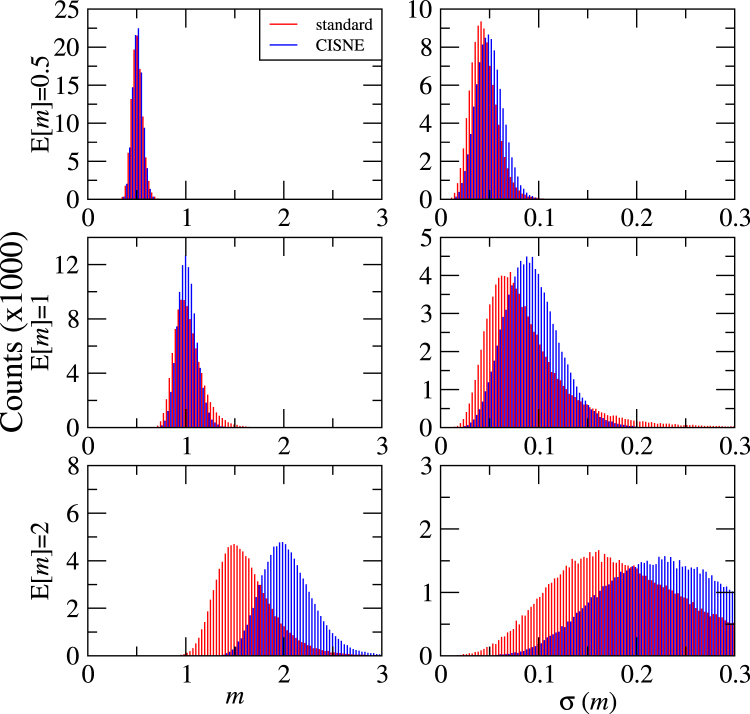
Results of 100000 Monte Carlo simulations for the parameter *m* and its standard deviation σ(*m*), obtained with the standard procedure and with the new method implemented in CISNE, for curves of different expected sigmoidality E[*m*].

The results of the fitting of the simulated data sets to the MEE are summarized in the histograms of Figs 4 and 5. In Fig. 4 we show the obtained values of *D* and its standard deviation σ(*D*). Notice that σ(*D*) is a measurement of the confidence interval of *D* from each of the experimental data sets separately, and it is not related to the standard deviation of the gaussian-like representations of *D* in the left panels of Fig. 4, which contain the information from all the experiments together. As expected, the obtained results for *D* follow a normal distribution centered at its expected value E[*D*] = 10. For the flat sigmoidal curve (*m* = 0.5), the results of the standard procedure and of the new CISNE method are very similar. This makes sense, as the selected data shown in Fig. 2(a) presents values of *f*_*a*_ which do not come very close to 0 or 1, and therefore almost all our data points stay in the central region of Fig. 2(c), where the change in the standard deviation of the points is not very important. However, the benefit of the new approach becomes apparent for hyperbolic (*m* = 1) and sigmoidal (*m* = 2) curves. This is accompanied by smaller values of σ(*D*). Therefore, CISNE allows to obtain values of *D* much closer to the real value, and with better confidence intervals, especially for *m* ≥ 1.

Figure 5 shows the obtained results for *m* and its standard deviation σ(m). The difference between the standard and the CISNE approach becomes even more clear in this case. CISNE presents gaussian distributions centered at the corresponding expected values E[*m*] = 0.5, 1 and 2, as expected. On the other hand, the standard procedure leads to a distribution with a maximum at *m* slightly lower than 1 for the hyperbolic curve and much lower than 2 for the sigmoidal curve. Moreover, the standard deviations *σ*(m) obtained with the standard procedure are lower than the ones obtained with the CISNE method (notice that in both cases σ(m) increases with E[*m*] = 0.5, but its relative value σ(m)/E[*m*] = 0.5 remains almost constant). Therefore, meanwhile CISNE reproduces accurately the results of *m*, the standard procedure can not only lead to wrong results, but could also lead to wrong confidence intervals which do not correspond to reality, leading to a false impression of accuracy in the results.

**Figure 6:**
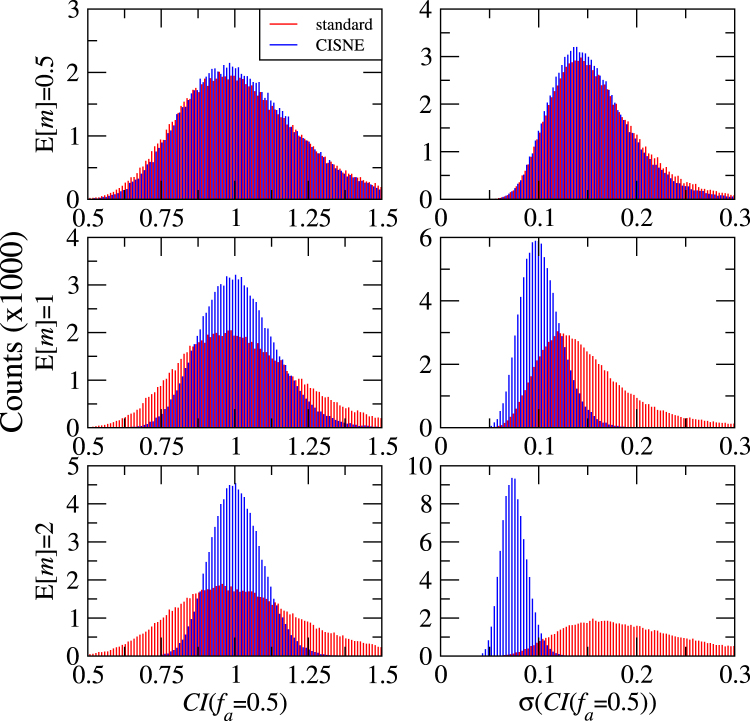
Results of 100000 Monte Carlo simulations for the combination index at *f*_*a*_ = 0.5 and its standard deviation σ(*D*), obtained with the standard procedure and with the new method implemented in CISNE, for curves of different expected sigmoidality E[*m*].

In order to analyse the accuracy of both methods in the determination of the *CI*, we perform blocks of 3 simulations with the same methodology as before, and we assign the first one to drug A, the second to drug B and the third to the drug combination A + B in a 1:1 ratio. As the 3 simulations have the same expected values of *m* and *D*, the obtained effect should be just additive and *CI*(*f*_*a*_) = 1. However, deviations from this value are expected from the effect of the experimental fluctuations. We repeat these blocks of simulations 100000 times for E[*m*] = 0.5, 1, 2 and the results of *CI*(*f*_*a*_ = 0.5) and its standard deviation σ(*CI*(*f*_*a*_ = 0.5)) are summarized in Fig. 6.

The results obtained with the standard and the new CISNE methods are similar for E[*m*] = 0.5, but the advantage of the new method is clear for E[*m*] = 1 and E[*m*] = 2. We show in Table 3 the percentage of the simulations that are inside an interval where we can consider synergistic (*f*_*a*_ < 0.8), additive (0.8 < *f*_*a*_ < 1.2), and antagonistic (1.2 < *f*_*a*_) effect. CISNE allows to strongly reduce the number of false positives of synergistic and antagonistic effect, especially for E[*m*] = 2, where the standard method leads to more than one third of simulations indicating non-additive effect, meanwhile this value is reduced to less than 3% of the experiments with the CISNE method. Notice that, as we are using a theoretical additive combination, wrong results appear as false synergistic or antagonistic results. However, the increase in the precision of the obtained *CI* with CISNE is not limited to reduce false positives, as shown in previous sections. If we consider a synergistic combination, the standard method can lead to wrong results of additive (or, even worse, antagonistic!) effects, and CISNE allows to minimize the probability of obtaining these wrong results as well.

**Table 3:** Percentage of our computational experiments of drug combinations that can be considered synergistic, additive or antagonistic based on the results of the standard and CISNE methods, and for different values of E[*m*].

E[*m*]	method	*CI*(*f*_*a*_ = 0.5) < 0.8 (synergistic)	0.8 < *CI*(*f*_*a*_ = 0.5) < 1.2 (additive)	1.2 < *CI*(*f*_*a*_ = 0.5) (antagonistic)
0.5	standard	13.47%	66.54%	19.99%
	CISNE	11.99%	69.86%	18.15%
1	standard	12.87%	67.69%	19.44%
	CISNE	3.73%	88.56%	7.72%
2	standard	15.11%	63.06%	21.84%
	CISNE	0.58%	97.37%	2.05%

## Conclusions

Combinations Therapies that present a synergistic effect is a valuable resource for the treatment of the most dreadful diseases. We have demonstrated in this article however that the accuracy whereby the available schemes determine whether a drug combination is synergistic or not can easily lead to false positives and false negatives. We have also identified the main source of inaccuracy and fixed it. Our new implementation allows us to determine dose-effect relationships more accurately, that conveys a more precise determination of the *CI*. In particular, our Monte Carlo simulations show that the number of false positives that can be obtained from the mathematical analysis can be reduced by a factor of 10. We find that the defects of the standard method become specially damaging whenever the experimental data deliver values of the fraction of the population affected *f*_*a*_ larger than 0.9 or smaller than 0.1. Although our new method presents advantages for any range of experimental data, we stress that the use of our new method becomes especially relevant in these cases.

## References

[1] Yuan R, Lin Y (2000). Traditional chinese medicine. Pharmacology & Therapeutics.

[2] Podolsky SH, Greene JA (2011). Combination drugs â€” hype, harm, and hope. New England Journal of Medicine.

[3] Bigioni M (2008). Antitumour effect of combination treatment with sabarubicin (men 10755) and cis-platin (ddp) in human lung tumour xenograft. Cancer Chemotherapy and Pharmacology.

[4] W. Humphrey R (2011). Opportunities and challenges in the development of experimental drug combinations for cancer. JNCI: Journal of the National Cancer Institute.

[5] Oversteegen L, Shah M, Rovini H (2007). Hiv combination products. Nat Rev Drug Discov.

[6] Breeze S (2012). Novel hiv-1 treatment stribild gains regulatory approval. Expert Review of Clinical Pharmacology.

[7] Mok TS (2017). Gefitinib plus chemotherapy versus chemotherapy in epidermal growth factor receptor mutation–positive non–small-cell lung cancer resistant to first-line gefitinib (impress): Overall survival and biomarker analyses. Journal of Clinical Oncology.

[8] Yang JC-H (2017). A review of regimens combining pemetrexed with an epidermal growth factor receptor tyrosine kinase inhibitor in the treatment of advanced nonsquamous Non-Small-Cell Lung cancer. Clinical Lung Cancer.

[9] Chen D, Liu X, Yang Y, Yang H, Lu P (2015). Systematic synergy modeling: understanding drug synergy from a systems biology perspective. BMC Systems Biology.

[10] Chen X (2016). Drug–target interaction prediction: databases, web servers and computational models. Briefings in Bioinformatics.

[11] Chen X (2016). Nllss: Predicting synergistic drug combinations based on semi-supervised learning. PLOS Computational Biology.

[12] Biosoft. Calcusyn, http://www.biosoft.com/w/calcusyn.htm (1997)

[13] Chou, T.-C. & Martin, N. Compusyn, http://www.combosyn.com (2006)

[14] Goldin A, Mantel N (1957). The employment of combinations of drugs in the chemotherapy of neoplasia: A review. Cancer Research.

[15] Greco WR, Bravo G, Parsons JC (1995). The search for synergy: a critical review from a response surface perspective. Pharmacological Reviews.

[16] Chou T-C (2006). Theoretical basis, experimental design, and computerized simulation of synergism and antagonism in drug combination studies. Pharmacological Reviews.

[17] Chou T-C (2010). Drug combination studies and their synergy quantification using the chou-talalay method. Cancer Research.

[18] Foucquier J, Guedj M (2015). Analysis of drug combinations: current methodological landscape. Pharmacol Res Perspect.

[19] Tallarida RJ (2011). Quantitative methods for assessing drug synergism. Genes Cancer.

[20] Tallarida RJ (2012). Revisiting the isobole and related quantitative methods for assessing drug synergism. Journal of Pharmacology and Experimental Therapeutics.

[21] Tallarida, R. J. *Drug Combinations: Tests and Analysis with Isoboles* (John Wiley & Sons, Inc., 2001). 10.1002/0471141755.ph0919s72 PMC4839184 26995550

[22] Chou T-C (1976). Derivation and properties of michaelis-menten type and hill type equations for reference ligands. Journal of Theoretical Biology.

[23] Chou T-C, Talalay P (1977). A simple generalized equation for the analysis of multiple inhibitions of michaelis-menten kinetic systems. Journal of Biological Chemistry.

[24] Chou T-C, Talalay P (1981). Generalized equations for the analysis of inhibitions of michaelis-menten and higher-order kinetic systems with two or more mutually exclusive and nonexclusive inhibitors. European Journal of Biochemistry.

[25] Chou T, Talalay P (1983). Analysis of combined drug effects: a new look at a very old problem. Trends in Pharmacological Sciences.

[26] Chou T-C, Talalay P (1984). Quantitative analysis of dose-effect relationships: the combined effects of multiple drugs or enzyme inhibitors. Advances in Enzyme Regulation.

[27] CISNE code for the identification of synergism numerically efficient, https://cisnecode.github.io (2017).

[28] Youssef MM (2016). Novel combination of sorafenib and biochanin-a synergistically enhances the anti-proliferative and pro-apoptotic effects on hepatocellular carcinoma cells. Scientific Reports.

[29] Seber, G. & Wild, C. *Nonlinear Regression*. Wiley Series in Probability an (Wiley, 2003).

[30] Metropolis N, Ulam S (1949). The monte carlo method. Journal of the American Statistical Association.

[31] Mackay, D. Introduction to monte carlo methods. In Jordan, M. (ed.) *Learning in Graphical Models*, chap. **6**, 175–204 (Springer Netherlands, 1998).

